# Impact of Free Delivery Care on Health Facility Delivery and Insurance Coverage in Ghana’s Brong Ahafo Region

**DOI:** 10.1371/journal.pone.0049430

**Published:** 2012-11-16

**Authors:** Susie Dzakpasu, Seyi Soremekun, Alexander Manu, Guus ten Asbroek, Charlotte Tawiah, Lisa Hurt, Justin Fenty, Seth Owusu-Agyei, Zelee Hill, Oona M. R. Campbell, Betty R. Kirkwood

**Affiliations:** 1 Faculty of Epidemiology and Population Health, London School of Hygiene and Tropical Medicine, London, United Kingdom; 2 Kintampo Health Research Centre, Ghana Health Service, Kintampo, Ghana; 3 Clinical Trials Unit, University of Nottingham, Nottingham, United Kingdom; 4 Institute of Child Health, University College London, London, United Kingdom; Aga Khan University, Pakistan

## Abstract

**Background:**

Many sub-Saharan countries, including Ghana, have introduced policies to provide free medical care to pregnant women. The impact of these policies, particularly on access to health services among the poor, has not been evaluated using rigorous methods, and so the empirical basis for defending these policies is weak. In Ghana, a recent report also cast doubt on the current mechanism of delivering free care – the National Health Insurance Scheme. Longitudinal surveillance data from two randomized controlled trials conducted in the Brong Ahafo Region provided a unique opportunity to assess the impact of Ghana’s policies.

**Methods:**

We used time-series methods to assess the impact of Ghana’s 2005 policy on free delivery care and its 2008 policy on free national health insurance for pregnant women. We estimated their impacts on facility delivery and insurance coverage, and on socioeconomic differentials in these outcomes after controlling for temporal trends and seasonality.

**Results:**

Facility delivery has been increasing significantly over time. The 2005 and 2008 policies were associated with significant jumps in coverage of 2.3% (p = 0.015) and 7.5% (p<0.001), respectively after the policies were introduced. Health insurance coverage also jumped significantly (17.5%, p<0.001) after the 2008 policy. The increases in facility delivery and insurance were greatest among the poorest, leading to a decline in socioeconomic inequality in both outcomes.

**Conclusion:**

Providing free care, particularly through free health insurance, has been effective in increasing facility delivery overall in the Brong Ahafo Region, and especially among the poor. This finding should be considered when evaluating the impact of the National Health Insurance Scheme and in supporting the continuation and expansion of free delivery care.

## Introduction

Millennium Development Goals 4 and 5– to reduce child mortality and improve maternal health – remain important global health challenges. Ensuring all women give birth with a skilled birth attendant and access to emergency obstetric care is accepted as the most crucial intervention for reducing maternal and newborn deaths [1], [2]. In Ghana, skilled attendance at delivery is unequally distributed: in 2003–2008 among the poorest 20% of women, 24% delivered with a health professional compared to 95% among the richest 20% [3]. Many factors can influence the rate of skilled birth attendance including the cost of care [4], which especially for emergency obstetric care can be catastrophic for households [5]. To address this issue, several countries in sub-Saharan Africa including Ghana have abolished fees for delivery care [6].

**Figure 1:**
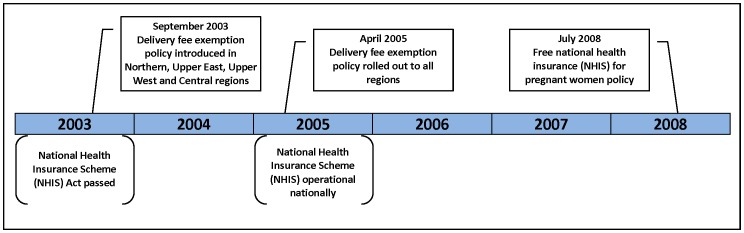
Timing of Ghana’s recent maternal health financing policies. Ghana introduced a delivery fee exemption policy in September 2003, which was rolled out to all regions in April 2005. This policy was followed by free national health insurance for pregnant women in July 2008.

In September 2003, the Government of Ghana attempted to increase skilled birth attendance and reduce inequality in use of services by introducing a policy exempting women in its four poorest regions from delivery care fees (Figure 1) [7]. In April 2005, this policy was rolled out to all regions. However, there were important problems with disbursing funding to health facilities and by October 2005 some regions had exhausted funds, resulting in some health facilities starting to charge clients again [7]. In 2003, the government also passed the National Health Insurance Scheme (NHIS) Act, although benefits were not accessible until 2005 [8]. The aim of the NHIS is to replace out-of-pocket fees at the point-of-service, as a more equitable health financing policy. Individuals pay an annual premium of about $10 USD, and although membership is mandatory, in practice many Ghanaians remain uninsured. In 2008, only 39% of women 15–49 years of age reported being insured, and among these 62% had a valid insurance card [3]. The level of NHIS coverage in the population as a whole, and the degree to which the poor in particular are covered, are an ongoing source of debate [9]–[11].

With funding for the 2003/2005 delivery fee exemption policy effectively running out around the same time as the NHIS was coming into effect, pregnant women who were not enrolled in the NHIS had to pay for maternity care. The Ministry of Health speculated that this may have decreased facility deliveries between 2006 and 2007 [12]. Subsequently, in July 2008, the government introduced its 2008 policy exempting pregnant women from paying the NHIS registration and premium fees. Enrolment entitles women to six antenatal visits, childbirth care (including complications), two postnatal visits within six weeks of childbirth, care of the newborn up to three months, and other primary health care benefits. All providers of maternity care services, including mission and private facilities, can participate in the NHIS.

Despite the importance of these two financing policies, there is limited evidence concerning their impact. Studies on the 2003/2005 policy (covering periods ranging from July 2002 to March 2006), reported an increase in facility births [13]–[15]. However, attribution to free care and effects on socioeconomic differentials in service utilization are equivocal, as temporal trends were not considered. To date, no studies have investigated the impact of the 2008 free NHIS policy in Ghana, and there is generally a “scarcity of good quality evidence” on the effect of such policies in low- and middle-income countries [16]. In addition, a 2011 report by Oxfam International and other non-governmental agencies suggested that Ghana’s NHIS as a whole is inefficient and unfair, as every Ghanaian pays for the NHIS through Value Added Tax, but coverage is low and skewed towards the richest [9].

A surveillance system covering all reproductive-aged women in seven Brong Ahafo Region districts between 2003 and 2010 provided a unique opportunity to examine whether the 2005 and 2008 policies increased health facility delivery and health insurance coverage, taking into account underlying secular trends, and to examine whether these policies benefited the poorest.

## Methods

### Data and Setting

Data were obtained from a health and demographic surveillance system supporting the ObaapaVitA [17] and Newhints [18] cluster randomized controlled trials (RCTs) carried out in seven contiguous predominantly rural districts in the Brong Ahafo Region (Kintampo North and South, Nkoranza North and South, Wenchi, Techiman, Tain). Information was gathered through four-weekly home visits by resident fieldworkers. Approximately 120,000 women of reproductive age live in this area, with about 18,000 pregnancies and 15,000 live births a year. We based our analysis on deliveries from January 2004 to December 2009: one year before the 2005 policy to one year after the 2008 policy was introduced. The 2003 policy did not apply to Brong Ahafo.

### Outcome Definitions

We examined trends in the percentage of deliveries taking place in a health facility, the percentage of delivered women enrolled with the NHIS, and socioeconomic differentials in both these outcomes.

Facility delivery included hospital, health centre and maternity home births. Data on NHIS enrolment were collected from March 2008, so analysis of insurance coverage was only possible from then until December 2009. Data were based on women’s self-reports collected at the first fieldworker visit after the birth. For 76% of women, this occurred within 30 days of the delivery. Socioeconomic differentials were examined using wealth quintiles (estimated from household asset data) and concentration indices which summarize how an outcome varies across the entire socioeconomic distribution. We restricted our analysis to records for which asset data had been collected within a year of the delivery, assuming assets would not change substantially within this timeframe. For women with multiple deliveries over the six-year period, data were collected for each delivery.

Principal component analysis was used to assign an asset score to each woman at the time of her delivery [19]. Reliability of these asset scores was confirmed by comparing them against individual asset ownership and educational levels of women. Women were then ranked from poorest to richest according to their scores, separately within each year of delivery from 2004 to 2009, and assigned to wealth quintiles, each representing a fifth (20%) of the women delivering within that year.

Concentration indices were calculated for facility delivery and insurance coverage by plotting the cumulative percent of each against the cumulative percent of women ranked by their asset scores, and calculating the area between this curve and the line of equality. This area by definition ranges from −1 to+1, with positive values corresponding to the curve being below the line of equality and the outcome concentrated towards the richest, and negative values where the curve is above the line and the outcome concentrated towards the poorest [20]. 95% confidence intervals for concentration indices were calculated using the bootstrap method [21].

### Temporal Trend Analysis

Monthly rates of facility delivery and insurance coverage were displayed graphically by wealth quintile using simple three-month moving averages [22]. We further studied temporal trends in these outcomes and in the monthly concentration indices of these outcomes using segmented linear regression models. We fitted separate temporal trends for three segments defined relative to the introduction of each policy: January 2004–March 2005, April 2005–June 2008, and July 2008–December 2009. We used a model of the form:
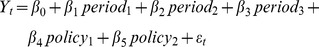
Where:

- Period_1_ is coded sequentially from 1 to 16 (April 2005 is 16^th^ month in study period) and remains 16 thereafter; β_1_ represents the trend from January 2004 to March 2005.
- Period_2_ is coded 0 until April 2005, then sequentially from 1 to 39 (July 2008 is 55^th^(16+39) month in study period) and 39 thereafter; β_2_ represents the trend from April 2005 to June 2008.
- Period3 is coded 0 until July 2008, then sequentially from 1 to 17 (December 2008 is 72^nd^(16+39+17) month in study period); β_3_ represents the trend from July 2008 to December 2009.
- Policy_1_ and policy_2_ are coded as indicator variables representing the two policies; their coefficients represent the immediate impact of the April 2005 policy and July 2008 policy, respectively.

The immediate impact of each policy was calculated as the absolute difference between the predicted values just before and after the policy. The longer-term impact was assessed by comparing temporal trends before and after each policy, calculated as the absolute difference between the regression slopes for each period. The models estimated the impact of each policy after controlling for temporal trends, and seasonal variation in facility delivery. We re-ran all models taking into account that some women contributed more than one delivery; however this adjusting for clustering at the woman-level did not significantly impact model parameters and was therefore not done in the final models. We also did not adjust for other measured determinants of facility delivery, such as rural residence or maternal education, as there was no evidence of change in their distribution during the study period.

We carried out all analysis using Stata version 11 [23].

## Results

### Sample Characteristics

Between January 2004 and December 2009, the surveillance system identified 92,462 deliveries. Of these, 91,015 women (98.4%) had complete data on assets and place of delivery and were included in the analysis of facility delivery. 27,841 women (99.8%) who delivered between March 2008 and December 2009 also had complete data on insurance enrolment and so were included in analysis of insurance coverage.

### Trend in Facility Delivery, January 2004–December 2009

**Figure 2:**
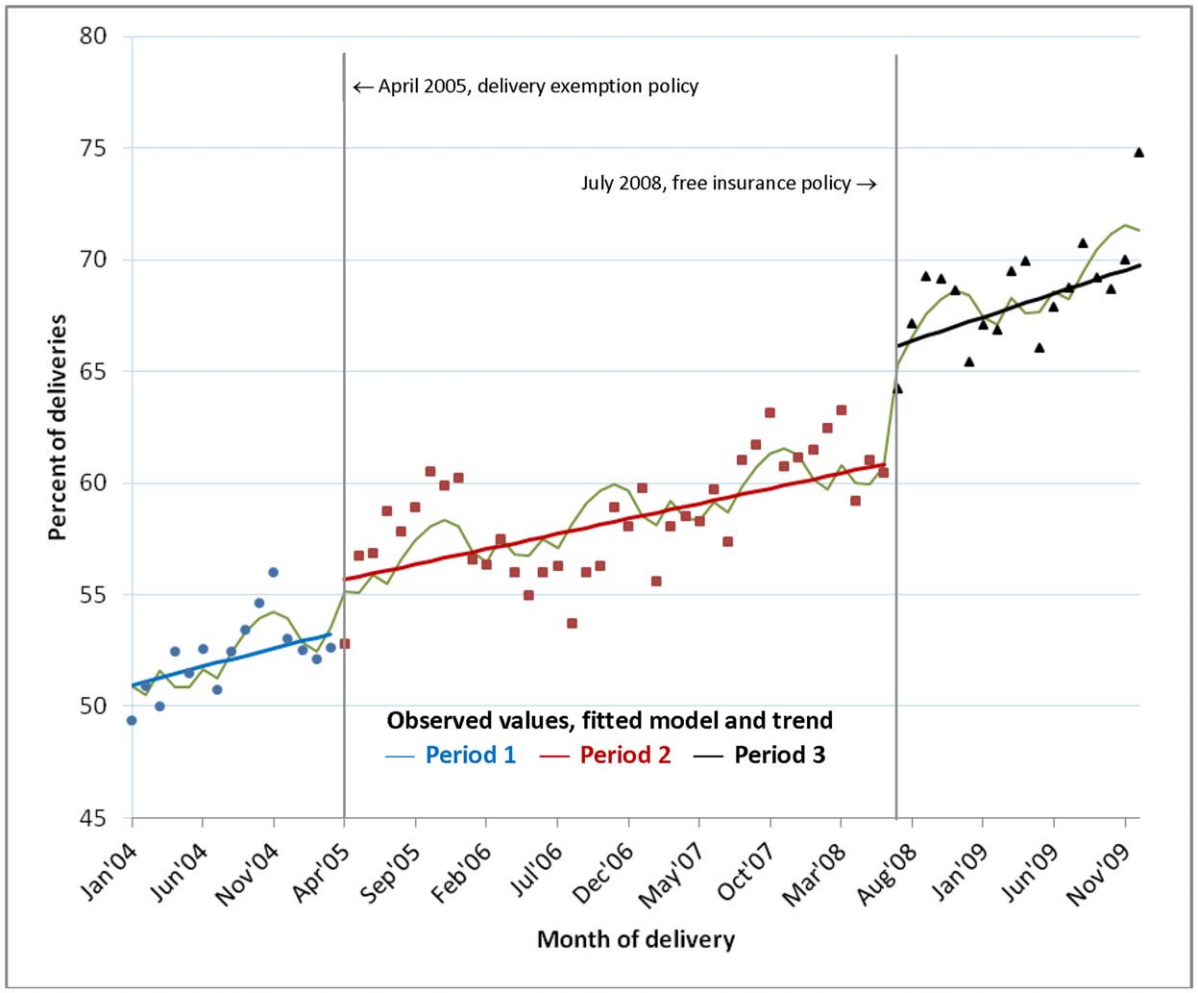
Facility deliveries, January 2004–December 2009: observed values, fitted model and trend. Figure shows the percentage of deliveries occurring in a facility each month in each policy period: observed values (dots), fitted model (wavy line) and trend (straight lines).

Figure 2 shows the observed percentage of women delivering in a health facility each month and the trend predicted from the fitted regression model, adjusted for month of delivery, temporal trend and policy change. There was an overall increase in facility delivery during this period, from 50.1% in January–March 2004 to 71.2% in October–December 2009. The regression results (Table 1) indicate an underlying temporal trend with statistically significant increases of 0.16%, 0.14% and 0.21% per month in the three time periods. These rates of increase were not significantly different across the three time periods. However, there was a statistically significant jump in coverage at the time of each policy. There were increases of 2.3% (p = 0.015) and 7.5% (p<0.001) after the 2005 free delivery care and 2008 free NHIS policies respectively, after adjusting for month of delivery and temporal trend.

**Table 1:** Trend in facility delivery, insurance coverage and inequality before and after April 2005 free delivery care and July 2008 free NHIS policies.

	Trend			Jump following policy		Change in trend	
	Period 1. January 2004–March 2005	Period 2. April 2005–June2008	Period 3. July 2008–December 2009	April 2005	July 2008	From Period 1 to 2	From Period 2 to 3
**Facility delivery** † **(% change per month)**							
	0.16	0.14	0.21	2.27	7.45	−0.03	0.07
	(−0.009,0.33)	(0.10,0.17)	(0.08,0.34)	(0.50,4.05)	(4.97,9.92)	(−0.20,0.15)	(−0.06,0.21)
	p = 0.063	p<0.001	p = 0.002	p = 0.015	p<0.001	p = 0.768	p = 0.281
**Concentration index facility delivery**							
	−0.0018	−0.00018	−0.0020	−0.0086	−0.0478	0.0016	−0.0018
	(−0.0019, −0.0018)	(−0.00019, −0.00017)	(−0.0020, −0.0019)	(−0.0092, −0.0080)	(−0.0485, −0.0470)	(0.0016,0.0017)	(−0.0018, −0.0018)
	p<0.001	p<0.001	p<0.001	p<0.001	p<0.001	p<0.001	p<0.001
**Insurance coverage** ‡ **(% change per month)**							
	NA	1.41	−0.14	NA	17.54	NA	−1.54
	NA	(0.52,2.29)	(−0.24, −0.04)	NA	(14.97,20.11)	NA	(−2.44, −0.65)
	NA	p = 0.002	p = 0.006	NA	p<0.001	NA	p = 0.001
**Concentration index insurance coverage**							
	NA	−0.0092	−0.0024	NA	−0.0890	NA	0.0070
	NA	(−0.0097, −0.0087)	(−0.0024, −0.0023)	NA	(−0.0903, −0.0876)	NA	(0.0063,0.0072)
	NA	p<0.001	p<0.001	NA	p<0.001	NA	p<0.001

† Facility delivery model is adjusted for month of birth.

‡ For insurance coverage, Period 2 corresponds to March 2008–June 2008.

**Figure 3:**
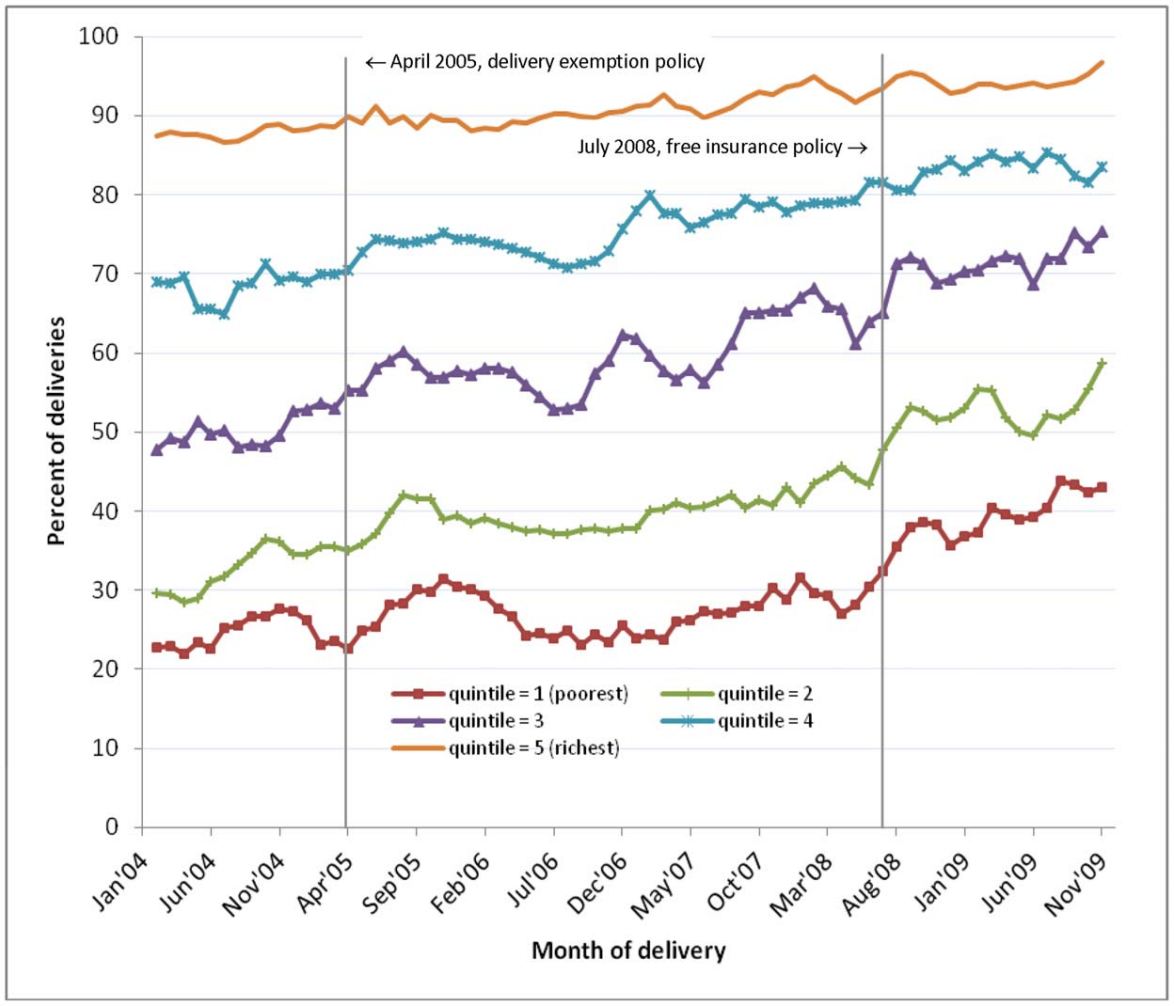
Facility deliveries, January 2004–December 2009: by wealth quintile (simple 3-month moving averages). Figure shows the percentage of deliveries occurring in a facility each month within each wealth quintile.

Figure 3 shows the trends in facility delivery by socioeconomic quintiles. Significant reductions in inequality were seen over time. The concentration indices in successive policy periods declined from 0.258 (95% CI: 0.251, 0.266) to 0.232 (95% CI: 0.227, 0.236) and to 0.173 (95% CI: 0.168, 0.178), with monthly declines observed in each policy period (Table 1). Socioeconomic inequality remained sizeable nonetheless. In January-March 2004, 64.7% more women in the richest quintile gave birth in a health facility compared with the poorest women (87.4% versus 22.7%). This difference declined to 53.8% by October-December 2009 (96.8% versus 43.0% respectively). Contributing to the overall declining inequality were relatively larger jumps in coverage among poorer than richer women after the July 2008 policy (Table 2). This pattern was not observed following the April 2005 policy.

**Table 2:** Immediate effect of April 2005 free delivery care and July 2008 free NHIS policies on facility delivery† and insurance coverage, by wealth quintile.

	Q1 (poorest)	Q2	Q3	Q4	Q5 (richest)	Overall
**Percentage increase in facility delivery in month of policy change**						
***April 2005 policy***	1.29	0.38	2.90	2.85	−0.36	2.27
	(−2.29,4.87)	(−3.58,4.35)	(−1.02,6.82)	(−0.62,6.32)	(−2.73,2.01)	(0.50,4.05)
	p = 0.480	p = 0.849	p = 0.147	p = 0.108	p = 0.766	p = 0.012
***July 2008 policy***	8.05	7.17	8.48	5.69	0.76	7.45
	(2.97,13.13)	(1.62,12.71)	(3.00,13.96)	(0.86,10.52)	(−2.50,4.03)	(4.97,9.92)
	p = 0.002	p = 0.011	p = 0.002	p = 0.021	p = 0.645	p<0.001
**Percentage increase in insurance coverage in month of policy change**						
***July 2008 policy***	30.59	28.70	13.11	8.49	5.81	17.54
	(24.04,37.14)	(22.37,35.04)	(7.55,18.67)	(3.52,13.46)	(2.17,9.46)	(14.97,20.10)
	p<0.001	p<0.001	p<0.001	p = 0.001	p = 0.002	p<0.001

† Facility delivery model is adjusted for month of birth.

### Trend in Insurance coverage, March 2008–December 2009

**Figure 4:**
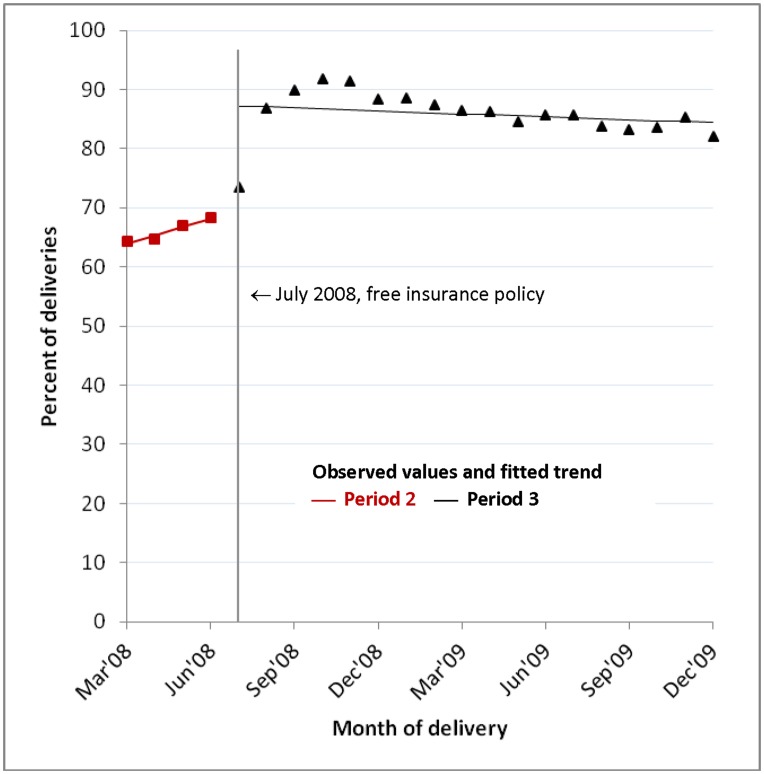
Insurance coverage, March 2008–December 2009: observed values and fitted trend. Figure shows the percentage of women with health insurance each month in each policy period: observed values (dots), fitted trend (straight lines).

Insurance coverage was rising at a rate of 1.4% (p = 0.002) per month before July 2008; it then jumped substantially (17.5%, p<0.001) after the free NHIS policy declining 0.14% per month (p = 0.006) after this (Table 1). This corresponds to 65.4% of women insured in March-May 2008 rising to a peak of 91.0% in September-November 2008 and declining to 83.7% by October-December 2009 (Figure 4). Coverage increased among women delivering in a health facility (from 81.1% to 86.5%), as well as among those delivering at home (from 40.9% to 77.8%) indicating that factors other than insurance status continued to influence women’s likelihood of a facility delivery.

**Figure 5:**
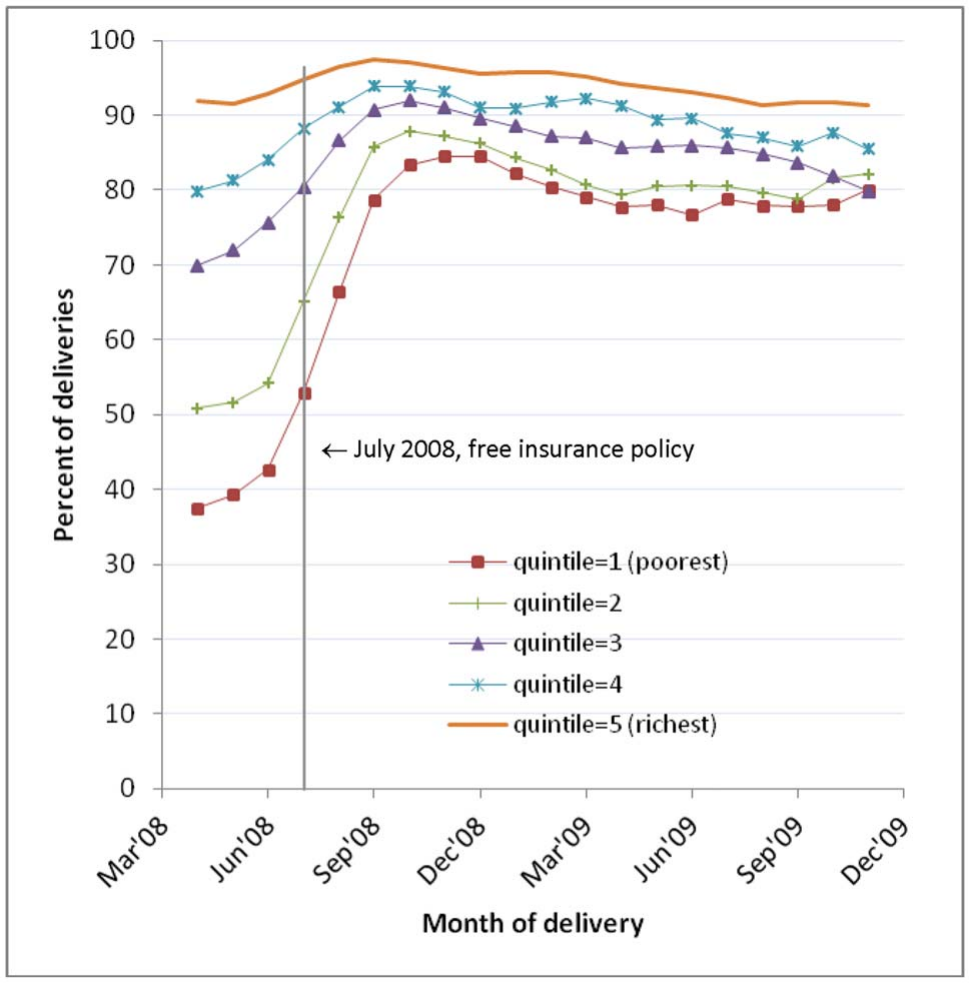
Insurance coverage, March 2008–December 2009: by wealth quintile (simple 3-month moving average). Figure 5 shows the percentage of women with health insurance each month within each wealth quintile.

Socioeconomic differentials in insurance coverage decreased significantly after the NHIS policy (Figure 5). In March-May 2008, 54.3% more women in the richest quintile compared to the poorest were insured. This difference decreased to 13.7% by September-November 2008, and to 11.4% by October-December 2009. The concentration index declined by 0.09 (p<0.001) in the month following the policy, compared to a decline of 0.009 per month in the months prior to the policy (Table 2). This accelerated reduction in inequality is evident in Figure 5 and is primarily a result of the larger immediate increases in coverage observed in poorer women compared with richer women (Table 2). The pre-policy concentration index for insurance coverage was 0.172 (95% CI: 0.161, 0.182) compared with 0.041 (95% CI: 0.038, 0.044) in the post-policy period.

## Discussion

Our study showed that in the seven study districts, facility delivery increased significantly over time and that there were statistically significant jumps of 2.3% and 7.5% in coverage following the 2005 and 2008 policies, respectively. In parallel, health insurance uptake showed a massive jump (of 17.5%) after the 2008 policy. We also found that increases were greatest among the poorest, and that consequently inequality in facility delivery and insurance coverage decreased. To our knowledge, this is the first study of the trends in facility delivery and insurance coverage associated with the free NHIS policy and only the second study to estimate the effect of removing user fees on delivery care after adjusting for temporal trends [24].

Our results are consistent with findings from a systematic review of user fee impacts which concluded that removing them increased utilization of services, usually in the form of one sharp step-up [16]. Our findings are also consistent with other studies in Ghana reporting cost as an important barrier to NHIS enrolment [3], [10], [25]. Other studies specific to delivery care also reported percentage point increases after fee removal [13], [14], [26], which may be due to reduced costs but also due to policies’ accompanying public health messages promoting facility delivery. Comparing our results to those of other studies is complicated by different contexts and by their lack of control for temporal trends making the effects attributable to fee removal uncertain. For example, evaluating Ghana’s 2005 delivery exemption policy Penfold et al reported a 5.0% increase in facility deliveries in the Volta Region [14], and Asante et al reported a 2.4% increase in the Volta and Central regions combined [13]._ENREF_13 Over the period studied by Penfold et al (Asante et al did not report their study period), our model predicts a 3.2% increase in the seven districts of which we estimated 2.3% (72% of the increase) could be attributable to the policy. The only other study reporting effects after adjusting for temporal trends, found no impact on institutional deliveries in Afghanistan which were already largely free (83.6%) prior to fee abolition [24].

It is noteworthy that a larger increase in facility delivery was observed around the time of the 2008 policy compared with the 2005 policy. Given the complex and multifactorial influences on facility delivery [4], factors contributing to this differential impact require further study. Areas of exploration could include the more comprehensive benefits under the NHIS policy, the act of enrolling for insurance promoting facility delivery, or well documented problems with implementation of the 2005 policy [7], [27], particularly the start-stop funding experienced by health facilities and ensuing informal costs. NHIS funding may be more reliable making informal costs less likely. Oxfam et al advocated replacing the NHIS architecture due to large-scale inefficiency; suggesting prospective payments to facilities instead of retrospective claims through the NHIS [9]. In light of the relative ineffectiveness of prospective financing with the 2005 delivery exemption policy, more careful examination of this recommendation is warranted.

It is also noteworthy that the NHIS policy was not only associated with increased facility delivery, but also reduced inequality in this outcome. Studies on benefit incidence by the World Bank have shown that the richest often benefit more than others when care is available free of charge because they are more able to express their demand and to influence healthcare professionals [28]. It is encouraging that this was not observed given the equity goals of the NHIS. The universality of the policy may have also contributed to promoting equality by avoiding the difficulties associated with identifying and targeting the poor with premium exemptions [29]. Oxfam et al urged the government to abolish NHIS premiums to reduce inequality in enrolment [9]. Our findings indicate that among pregnant women abolishing premiums greatly reduced such inequality.

In our study, 65.4% of delivering women in March-May 2008 reported NHIS enrolment. Comparatively in 2008, 59% of 15–49 year old women in the Brong Ahafo Region reported enrolment, of whom 57% showed valid NHIS cards indicating that at least 34% (57% of 59%) had active coverage [3]. These coverage rates are much higher than the 18% active coverage Oxfam et al estimated for the population as a whole in 2009 [9]. The reasons for the lower Oxfam estimate are uncertain, but may be partly because it applies to the whole population. Even prior to free NHIS coverage, pregnant women may have been more incentivised to enrol in anticipation of requiring medical care during pregnancy for themselves or for their newborn.

Following the NHIS policy, there were larger increases in insurance coverage than facility delivery, suggesting greater complexity in the determinants of facility delivery than insurance coverage. For example, insurance enrolment is more feasible with a one-year window covering the pregnancy, delivery and postpartum period in which to act, compared to the short and unpredictable window associated with labour and delivery. Another factor may be the perceived benefit of insurance versus that of facility delivery. NHIS benefits are probably desired by most women, while perception of the benefits of skilled attendance are more variable, depending on factors such as awareness of the dangers of childbirth, social barriers and past pregnancy experiences [4], [30]. _ENREF_4The greater reduction in inequality in insurance coverage than in inequality in facility births also reflects other barriers to seeking care being disproportionally higher in the poor. In 2008, 50% of women in the lowest wealth quintile reported transportation to a health facility as a serious problem, compared with 13% of women in the highest wealth quintile [3].

Our study has a number of limitations. Due to its ecological design, we cannot rule out the possibility that factors besides free care policies led to these findings. However, we are not aware of any other contextual issues that may explain these results and hence, we believe that the assumption that the pre-existing trends would have continued without each policy is a reasonable one on which to base our analysis. Data were not collected on the timing of NHIS enrolment, so it was not possible to determine whether insurance status prior to delivery was associated with the place of birth. Finally, we recognize that increased facility delivery does not necessarily mean better maternal and newborn health outcomes, as this is a factor of the quality of care available. Further research is needed to determine health impacts.

### Conclusion

This was the first study examining the effects of Ghana’s 2008 free NHIS policy on facility delivery, insurance coverage and inequality in these two outcomes. The results suggest that the 2008 policy was associated with large increases in insurance coverage and increases in facility delivery. It was also followed by reductions in inequality in these outcomes. Notwithstanding these findings, free delivery care needs to be part of a multipronged approach that addresses other barriers to accessing delivery care, especially those experienced by the poor. These finding should be considered when evaluating the impact of the NHIS and in supporting the continuation and expansion of free delivery care.
